# Increased Extravascular Lung Water Reduces the Efficacy of Alveolar Recruitment Maneuver in Acute Respiratory Distress Syndrome

**DOI:** 10.1155/2012/606528

**Published:** 2012-05-08

**Authors:** Alexey A. Smetkin, Vsevolod V. Kuzkov, Eugeny V. Suborov, Lars J. Bjertnaes, Mikhail Y. Kirov

**Affiliations:** ^1^Department of Anesthesiology and Intensive Care Medicine, Northern State Medical University, Troitsky Avenue 51, Arkhangelsk 163000, Russia; ^2^Department of Anesthesiology and Intensive Care Medicine, City Hospital #1 of Arkhangelsk, Suvorov Street 1, Arkhangelsk 163001, Russia; ^3^Department of Clinical Medicine (Anesthesiology), Faculty of Health Sciences, University of Tromsø, MH-Breivika, Tromsø 9038, Norway

## Abstract

*Introduction*. In acute respiratory distress syndrome (ARDS) the recruitment maneuver (RM) is used to reexpand atelectatic areas of the lungs aiming to improve arterial oxygenation. The goal of our paper was to evaluate the response to RM, as assessed by measurements of extravascular lung water index (EVLWI) in ARDS patients. *Materials and Methods*. Seventeen adult ARDS patients were enrolled into a prospective study. Patients received protective ventilation. The RM was performed by applying a continuous positive airway pressure of 40 cm H_2_O for 40 sec. The efficacy of the RM was assessed 5 min later. Patients were identified as responders if PaO_2_/FiO_2_ increased by >20% above the baseline. EVLWI was assessed by transpulmonary thermodilution before the RM, and patients were divided into groups of low EVLWI (<10 mL/kg) and high EVLWI (≥10 mL/kg). *Results*. EVLWI was increased in 12 patients. Following RM, PaO_2_/FiO_2_ increased by 33 (4–65) % in the patients with low EVLWI, whereas those in the high EVLWI group experienced a change by only −1((−13)–(+5)) % (*P* = 0.035). *Conclusion*. In ARDS, the response to a recruitment maneuver might be related to the severity of pulmonary edema. In patients with incresed EVLWI, the recruitment maneuver is less effective.

## 1. Introduction

The consolidation of pulmonary tissue and, in particular, the formation of atelectases is a key component in the pathogenesis of acute lung injury (ALI) and its most severe form, acute respiratory distress-syndrome (ARDS) [1]. Loss of pulmonary tissue aeration resulting from decreased production of surfactant, evolvement of lung edema, and denudation of alveolar basal membrane, is one of the crucial mechanisms of intrapulmonary shunting and arterial hypoxemia [2]. The formation of atelectases can also be triggered by gravity forces related to the increased weight of the edematous parts of the lungs resulting in a fall in functional residual capacity and compression of dependent lung areas in the supine patient [1].

The accumulation of interstitial, alveolar, and migrating cellular fluid in the lungs may also play an important role in the pathogenesis of ARDS, although its importance is often underestimated [3, 4]. Obviously, in severe lung edema the lung fluid content, which is reflected by extravascular lung water, can increase 2-3-fold prior to a significant decrease in arterial oxygenation [5]. Increments in extravascular lung water content of 500–700 mL up to 1000–1800 mL, corresponding to increments in extravascular lung water index (EVLWI) of from 7–10 mL/kg to 14–25 mL/kg may be seen. An experimental study from our group demonstrated that such an increase in EVLWI is not necessarily accompanied by a substantial expansion of the pulmonary parenchyma, as assessed by spiral computer tomography (CT) [6]. The expansion of the extravascular fluid volume may take place at the expense of a compression in the conducting airways and alveoli and, to a minor extent, of the vascular bed, since severe pulmonary hypertension is not a prerequisite for the evolvement of ARDS [7]. Most likely accumulation of extravascular lung water in the early exudative phase of ARDS may result in destabilization of alveolar tissue requiring higher PEEP values to counteract gravity-related lung collapse and consolidation.

The aim of the alveolar recruitment maneuver (RM) is to expand and reopen collapsed lung tissue by intermittent short-acting increase in airway pressure. In the general ICU population, RM may improve the oxygenation ratio (PaO_2_/FiO_2_) by 29–50% of ARDS patients [8–10]. However, this method also has a number of side-effects and complications, the most severe being barotrauma and compromised cardiac preload [11, 12]. Notably, these adverse effects are more pronounced in nonresponders with a considerable decrease in the individual benefit-to-risk ratio [13].

Therefore, an active search for predicting an individual's response to RM seems to be reasonable. Assuming there is a potential propensity of edematous pulmonary tissue to consolidate, or vice versa, a resistance of injured parenchyma to reopen, we hypothesized that EVLWI may influence the efficacy of the recruitment maneuver in ARDS patients. Thus, the aim of our study was to evaluate the response to RM, as assessed by EVLWI, in patients with ARDS.

## 2. Materials and Methods

The study was approved by the Medical Ethics Committee of Northern State Medical University, Arkhangelsk, Russian Federation. Written informed consent was obtained from every patient or his/her next of kin.

This prospective pilot study was performed in a 900-bed university hospital. From 2007 to 2010, we enrolled 17 adult patients who met the ALI/ARDS criteria according to the American European Consensus Conference [14]. Exclusion criteria were duration of ALI/ARDS >24 hrs, hypovolemia, severe COPD, and/or severe cerebral or cardiac diseases.

Patients were sedated with fentanyl (1 mcg/kg/hr) and midazolam (0.05 mg/kg/hr) and ventilated using pressure-controlled ventilation (PCV) (Avea, Viasys, USA) with the following initial settings: FiO_2_ 0.5, positive end-expiratory pressure (PEEP) 5 cm H_2_O, driving pressure to a targeted tidal volume of 7 mL/kg of predicted body weight (PBW), and a respiratory rate providing a PaCO_2_ of 35–45 mm Hg. For males, PBW (kg) was calculated as = 50 + 2.3 (height (cm)/2.54–60), and correspondingly for females PBW (kg) = 45 + 2.3 (height (cm)/2.54–60). If the initial ventilator settings did not result in a SaO_2_ ≥94% and/or PaO_2_ ≥70 mm Hg, FiO_2_ was increased in steps of 0.1 up to 0.8 and remained unchanged during the study.

Hemodynamic monitoring was performed using the single transpulmonary thermodilution technique. In all patients the femoral artery was cannulated with a 5F thermodilution artery catheter (Pulsiocath PV2015L20, Pulsion). The catheter was connected to a PiCCO*plus* (Pulsion Medical Systems, Germany) monitor for measurements of cardiac index (CI), extravascular lung water index (EVLWI, which was adjusted to PBW), global end-diastolic volume index (GEDVI), systemic vascular resistance index (SVRI), mean systemic arterial pressure (MAP), and heart rate (HR). The thermodilution measurements were performed in triplicate with injections of ice-cold (<8°C) 5% dextrose solution via a preinserted jugular central venous catheter (8.5F triple-lumen 20 cm catheter).

After initial measurements and muscular relaxation with pipecuronium (0.06 mg/kg), RM was performed by subjecting the patients to a continuous positive airway pressure of 40 cm H_2_O for a period of 40 seconds [10]. The RM was discontinued in case of hypotension (MAP <50 mm Hg or a decrease in MAP of more than 30 mm Hg from the initial value), or hypoxemia (SpO_2_ <85% or a decrease of more than 10%). Then PCV was resumed with the same settings as before the RM. PEEP was set at 2 cm H_2_O above the lower inflection point (LIP) of the pressure-volume (*P-V*) curve determined by an inflection point maneuver by the ventilator (Avea, Viasys, USA). The efficacy of the recruitment maneuver was assessed by registering the change in PaO_2_/FiO_2_ five minutes later. Patients were identified as responders if PaO_2_/FiO_2_ increased by at least 20% [8, 10, 13]. The stability of RM was assessed by following changes in PaO_2_/FiO_2_ at 40–60 min after the return to PCV.

For additional analysis of the efficacy of RM, patients were divided by the baseline EVLWI values as low EVLWI (<10 mL/kg) and high EVLWI (≥10 mL/kg) groups [4, 15].

Hemodynamic parameters were evaluated at baseline. Blood gases, lung mechanics, and parameters of mechanical ventilation were registered before RM and at 5 min and 40–60 min after RM.

### 2.1. Statistical Analysis

For data collection and analysis we used SPSS software (version 18.0; SPSS Inc., Chicago, IL, USA). Power analysis was not performed because of the pilot design of the study. The data distribution was assessed with Shapiro-Wilk's test. Quantitative data were presented as mean ± standard deviation or median (25th–75th percentile) depending on the data distribution. Discrete data were expressed as absolute values or percentages. In case of normal distribution, we used two-tailed Student's *t*-test for comparisons between the groups and repeated measures *t*-test for assessment of intragroup changes. Nonparametrically distributed data were assessed by two-tailed Mann-Whitney's *U*-test and Wilcoxon's test for comparisons between and within the groups, respectively. Discrete data were evaluated using Fisher's exact test. For all tests a *P* value <0.05 was considered significant.

## 3. Results

Fourteen male and three female patients were enrolled into the study. The mean age of the patients was  47 ± 2 yrs. In most cases (94%), the baseline PaO_2_/FiO_2_ was less than 200 mm Hg.

### 3.1. The Efficacy of the Recruitment Maneuver: Responders and Nonresponders

The recruitment maneuver was accompanied by an increase in PaO_2_/FiO_2_ of more than 20% of the baseline value in 5 patients (*responders*) and did not affect oxygenation significantly in 12 patients (*nonresponders*). The demographic characteristics of responders and nonresponders are presented in Table 1. The groups did not differ regarding age, weight and height, type of ARDS, and the severity of lung injury or other organ dysfunctions. Baseline PaO_2_/FiO_2_ values were similar in both groups (Table 2).

The RM increased PaO_2_/FiO_2_ by a median of 62 (32–91) % in the responders, whereas the nonresponders demonstrated no changes or even decreased PaO_2_/FiO_2_ compared to the baseline value: 1((−13)–(+4))% (*P* = 0.002). Despite improvement in PaO_2_/FiO_2_ after RM in the responders, the PaO_2_/FiO_2_ did not differ significantly between responders and nonresponders (Table 2).

The stability of the RM was evaluated in 12 patients including 4 responders and 8 nonresponders. A decrease in PaO_2_/FiO_2_ of more than 15% compared with values yielded immediately after recruitment was found in 58% of patients including 75% of the responders and 38% of the nonresponders. The average decreases in PaO_2_/FiO_2_ were 61 (6–102) % and 14 (4–22) % in responders and nonresponders, respectively (*P* = 0.19). Hemodynamics and ventilatory variables did not differ significantly between responders and nonresponders (Table 2).

### 3.2. Association between the Efficacy of the Recruitment Maneuver and Extravascular Lung Water

Increased EVLWI (≥10 mL/kg) was found in 12 patients including two responders (40% of all responders) and 10 nonresponders (83% of all nonresponders). EVLWI did not differ between patients with direct and indirect ARDS.

The general characteristics of patients with low and high EVLWI are presented in Table 3. Patients with low EVLWI had higher SOFA score values (Table 3).

**Figure 1:**
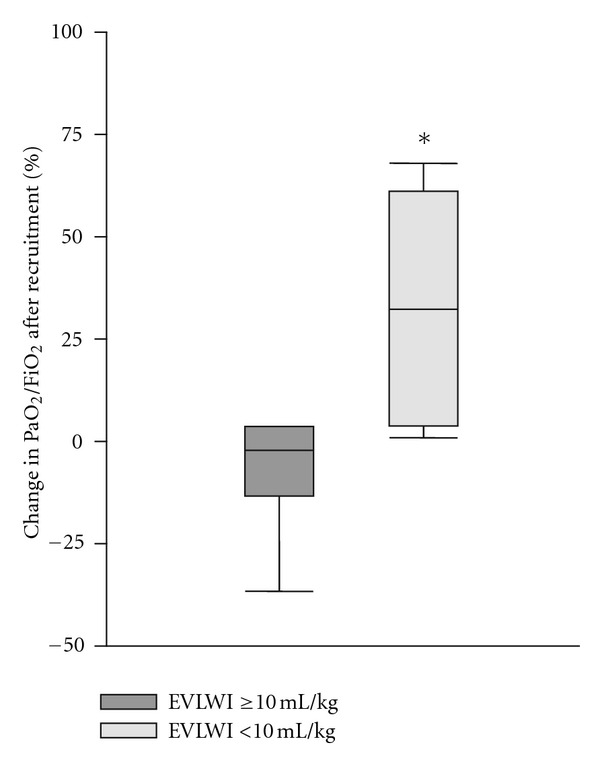
Changes in PaO_2_/FiO_2_ following recruitment maneuver in patients with increased (>10 mL/kg) and low (<10 mL/kg) extravascular lung water indexes. EVLWI: extravascular lung water index. *P < 0.05 between the groups (Mann-Whitney's test).

The baseline PaO_2_/FiO_2_ did not differ between patients with low and high EVLWI. In response to the RM patients in the low EVLWI group demonstrated a 33 (4–65) % increase in PaO_2_/FiO_2_. In contrast patients with EVLWI ≥10 mL/kg showed no substantial changes in PaO_2_/FiO_2_: −1((−13)–(+5)) (*P* = 0.035 compared with the low EVLWI group) (Figure 1).

During the assessment of recruitment stability, PaO_2_/FiO_2_, PaCO_2_, and hemodynamic parameters were similar in patients with low and increased EVLWI (Table 4). Baseline tidal volume was higher in the low compared to the high EVLWI group.

## 4. Discussion

 Our study demonstrates that during ALI and ARDS the efficacy of alveolar recruitment depends, at least partly, on the content of extravascular lung water. Pulmonary edema is associated with a reduced capability of 40 cm H_2_O × 40 sec RM to improve arterial oxygenation, thus, necessitating a search for other interventions to counteract hypoxemia during ARDS.

Alveolar RM is an important component of the open lung strategy in patients with ALI/ARDS of different etiologies. There are multiple modifications of the RM technique with individual adverse effects and benefits [16–18]. One extensively used principle is to increase pressure in the airways related to the consolidated areas over the level of the re-opening pressure [19]. A short-term sustained inflation pressure of up to 40 cm H_2_O for 40 seconds is the simplest and most well-studied version of RM, commonly used in ARDS patients.

Our study showed that 40 cm H_2_O × 40 sec RM resulted in a substantial improvement in PaO_2_/FiO_2_ in 29% of the patients. This is consistent with the findings of other recent investigators who reported the percentage of responders as 29–50% [8–10]. It is intriguing that the PaO_2_/FiO_2_ in responders and nonresponders was similar after the RM but the difference in response can be explained by the tendency to lower baseline PaO_2_/FiO_2_ in responders. In 75% of the responders and 38% of the nonresponders PaO_2_/FiO_2_ decreased within 40–60 minutes following the RM despite having identified and set an optimal individual PEEP value (2 cm H_2_O above LIP of the *P-V* curve). Indeed, the effect of alveolar recruitment is unstable; PaO_2_/FiO_2_ may decrease to baseline values as quickly as 30–45 minutes after PEEP has been adjusted [20, 21]. The stability of the alveolar reexpansion may be limited by the technique used to detect the optimal PEEP. The adjustment of an optimal PEEP using the pressure-volume (*P-V*) curve, as used in this study, is probably one of the most widespread and preferable methods for use at the bedside [22]. However, particularly in patients with “stiff” lungs resulting from severe ARDS, the lower inflection point of the *P-V* curve may be hard to discern [23].

The response to an RM may be affected by a wide range of factors, including the origin of ALI (direct or indirect), the technique used for the recruitment and the PEEP level used to maintain the patency of the airways following the forced reexpansion [24]. However, the effects are still controversial. Several studies demonstrate that indirect ALI/ARDS may be associated with a decreased response to RM [25, 26], while others disagree with these assumptions [10, 27]. In the present study no association was found between the type of ARDS and the response to RM.

Increased interstitial hydrostatic pressure and pulmonary weight have been suggested to be among the key mechanisms of atelectasis formation in ALI/ARDS according to the “sponge theory,” postulating a fall in lung compliance combined with compression and collapse of dependent small airways [24, 28, 29]. Studies carried out with the use of spiral CT have revealed that RM can lead to overdistension of intact or minimally injured areas located adjacent to the consolidated foci of lung tissue, resulting in volume- and/or biotrauma [30]. In areas of collapsed and consolidated lung tissue, particularly in regions of focal deaeration, a RM of 40 cm H_2_O does not regularly result in a substantial improvement in aeration [13, 29–31].

In this study, patients with low EVLWI (<10 mL/kg) showed a significant increase in PaO_2_/FiO_2_ following RM. In contrast, those with pulmonary edema failed to respond with an improvement in arterial oxygenation. However, we found no significant correlation between EVLWI and the percentage of positive response to RM. The cut-off value for EVLWI of 10 mL/kg was selected according to the results obtained by Chung and coauthors, who demonstrated that EVLWI ≥10 mL/kg predicts mortality with a sensitivity of 94.7% and a specificity of 66.7% [4]. In our study, EVLWI was above 10 mL/kg PBW in 71% of patients. This is in agreement with previously published data from our group [32]. In addition, according to the above definition, EVLWI was increased in 40% of the responders and 83% of the nonresponders. Indeed, pulmonary edema and aeration of lung parenchyma are closely associated. Extravascular lung water index correlates with the CT-reconstructed volume of pulmonary tissue of aqueous density, both in experimental [6] and clinical settings [33]. However, the accuracy of EVLWI measurement might be influenced by pulmonary vascular obstruction and prevalence of focal or regional pulmonary injury [34]. In the absence of lung edema, the atelectatic areas might be more compliant to the transiently increased airway pressure, similar to compression atelectasis where gas remains in the occluded acinar compartment [35].

Our study has several limitations, first of all, a small sample size. Thus, further larger studies of extravascular lung water and alveolar recruitment are warranted. The numerical differences in mean tidal volumes between the groups may be explained by different predicted body weights and dynamic ventilatory properties of the edematous and nonedematous lungs. Surprisingly, in this population of critically ill patients, the SOFA score was higher in the group with low EVLWI. This finding may confirm our assumption that the severity of pulmonary edema rather than dysfunction of other organs is a key factor that might affect the efficacy of the RM in ARDS patients.

## 5. Conclusions

In ALI and ARDS responses to the lung recruitment maneuver (40 cm H_2_O × 40 sec) may depend on the severity of pulmonary edema. In patients with EVLWI above 10 mL/kg, the recruitment maneuver may be less effective and may even be considered as contraindicated.

## Figures and Tables

**Table 1:** General characteristics of responders and nonresponders to lung recruitment maneuver.

Parameter	Responders (*n* = 5)	Nonresponders (*n* = 12)	*P*
Age, years	44.2 ± 16.7	47.6 ± 17.2	0.71
Gender, male/female	5/0	9/3	0.50
Height, cm	178 ± 4	172 ± 8	0.11
Actual body weight, kg	84.6 ± 20.8	77.0 ± 12.1	0.35
Predicted body weight, kg	73.3 ± 3.2	66.5 ± 7.2	0.06
Type of ARDS, direct/indirect	4/1	8/4	1.00
SAPS II, points	40.0 ± 12.4	44.6 ± 14.9	0.56
SOFA, points	9.0 ± 3.2	7.9 ± 2.6	0.47
Murray score, points	2.50 (2.25–3.08)	2.25 (2.06–2.75)	0.52

Data are presented as mean ± standard deviation, absolute values or median (25th–75th percentile).

**Table 2:** Arterial blood gases, hemodynamics and parameters of mechanical ventilation in responders and nonresponders to lung recruitment maneuver.

Parameter	Responders (*n* = 5)	Nonresponders (*n* = 12)	*P*
PaO_2_/FiO_2_ baseline, mm Hg	127 ± 50	155 ± 45	0.27
PaO_2_/FiO_2_ after RM, mm Hg	158 (136–311)	152 (116–161)	0.29
PaO_2_/FiO_2_ stability of RM, mm Hg	152 ± 63	141 ± 44	0.71
PaCO_2_ baseline, mm Hg	45 ± 8	45 ± 8	0.98
PaCO_2_ after RM, mm Hg	45 ± 12	49 ± 8	0.25
PaCO_2_ stability of RM, mm Hg	43 ± 7	48 ± 8	0.27
CI, L/min/m^2^	3.17 ± 0.90	3.84 ± 1.29	0.31
MAP, mm Hg	71 ± 7	96 ± 26	0.06
SVRI, dyn sec cm^−5^/m^2^	1717 (1089–1994)	1662 (1285–2271)	0.46
HR, beat/min	95 ± 8	112 ± 29	0.22
GEDVI, mL/m^2^	702 ± 136	695 ± 130	0.92
EVLWI, mL/kg	11.6 ± 5.5	13.1 ± 4.4	0.55
FiO_2_, %	50 (50–80)	50 (50–60)	0.51
Tidal volume, mL	494 ± 58	444 ± 55	0.14
Minute ventilation, L/min	11.6 ± 4.2	10.3 ± 1.6	0.43
Dynamic respiratory compliance, mL/cm H_2_O	29 (26–62)	28 (24–35)	0.39

Data are presented as mean ± standard deviation or median (25th–75th percentile).

RM: recruitment maneuver; CI: cardiac index; MAP: mean arterial pressure; SVRI: systemic vascular resistance index; HR: heart rate; GEDVI: global end-diastolic volume index; EVLWI: extravascular lung water index.

**Table 3:** General characteristics of patients with low and increased extravascular lung water index.

Parameter	EVLWI <10 mL/kg (*n* = 5)	EVLWI ≥10 mL/kg (*n* = 12)	*P*
Age, years	40.4 ± 14.9	49.2 ± 17.2	0.34
Gender, male/female	5/0	9/3	0.52
Type of ARDS, direct/indirect	3/2	9/3	0.60
SAPS II, points	38 ± 7	46 ± 16	0.29
SOFA, points	10.4 ± 2.7	7.3 ± 2.3	**0.03**
Murray score, points	2.50 (2.38–2.75)	2.25 (2.06–3.12)	0.36

Data are presented as mean ± standard deviation, absolute values or median (25th–75th percentile).

**Table 4:** Blood gases, hemodynamics, and parameters of mechanical ventilation in patients with low and increased extravascular lung water index.

Parameter	EVLWI <10 mL/kg (*n* = 5)	EVLWI ≥10 mL/kg (*n* = 12)	*P*
PaO_2_/FiO_2_ at baseline, mm Hg	117 ± 34	159 ± 47	0.09
PaO_2_/FiO_2_ after RM, mm Hg	146 (122–177)	158 (122–168)	0.46
PaO_2_/FiO_2_ stability of RM, mm Hg	134 ± 58	149 ± 46	0.58
Changes in PaO_2_/FiO_2_ within the period of stability assessment, %	−14((−1)–(−5))	−18((−37)–(−9))	0.68
PaCO_2_ at baseline, mm Hg	45 ± 8	45 ± 8	0.89
PaCO_2_ after RM, mm Hg	49 ± 13	48 ± 9	0.54
PaCO_2_ stability of RM, mm Hg	43 ± 6	48 ± 8	0.29
CI, L/min/m^2^	3.61 ± 0.98	3.65 ± 1.32	0.95
MAP, mm Hg	75 (66–106)	88 (71–99)	0.40
SVRI, dyn sec cm^−5^/m^2^	1717 (1089–2144)	1597 (1285–2238)	0.75
HR, beat/min	101 (97–105)	103 (84–133)	0.92
GEDVI, mL/m^2^	654 ± 92	714 ± 140	0.39
EVLWI, mL/kg	8.2 (6.0–9.1)	15.8 (11.2–17.8)	**0.002**
FiO_2_, %	50 (50–80)	50 (50–60)	0.51
Tidal volume, mL	504 ± 34	439 ± 58	**0.04**
Minute ventilation, L/min	11.6 (11.4–14.6)	9.9 (8.4–12.0)	0.06
Dynamic respiratory compliance, mL/cm H_2_O	29 (26–59)	28 (24–35)	0.67

Data are presented as mean ± standard deviation, absolute values or median (25th–75th percentile).

RM: recruitment maneuver; CI: cardiac index; MAP: mean arterial pressure; SVRI: systemic vascular resistance index; HR: heart rate; GEDVI: global end-diastolic volume index; EVLWI: extravascular lung water index.
